# Association of Fluid Balance and Survival of Pediatric Patients Treated With Extracorporeal Membrane Oxygenation

**DOI:** 10.3389/fped.2021.722477

**Published:** 2021-09-16

**Authors:** Prakadeshwari Rajapreyar, Lauren Castaneda, Nathan E. Thompson, Tara L. Petersen, Sheila J. Hanson

**Affiliations:** ^1^Department of Pediatric Critical Care, Medical College of Wisconsin, Milwaukee, WI, United States; ^2^Children's Wisconsin, Milwaukee, WI, United States; ^3^Children's Hospital of Colorado, Colorado Springs, CO, United States

**Keywords:** fluid balance, extracorporeal membrane oxygenation, mechanical circulatory support, fluid overload on ECMO, survival on ECMO

## Abstract

The effect of positive fluid balance (FB) on extracorporeal membrane oxygenation (ECMO) outcomes in pediatric patients remains unknown. We sought to evaluate if positive FB in pediatric intensive care unit (PICU) patients with respiratory and/or cardiac failure necessitating ECMO was associated with increased morbidity or mortality. This was a multicenter retrospective cohort study of data from the deidentified PEDiatric ECMO Outcomes Registry (PEDECOR). Patients entered into the database from 2014 to 2017, who received ECMO support, were included. A total of 168 subjects met the study criteria. Univariate analysis showed no significant difference in total FB on ECMO days 1–5 between survivors and non-survivors [median 90 ml/kg (IQR 18–208.5) for survivors vs. median 139.7 ml/kg (IQR 11.2–300.6) for non-survivors, *p* = 0.334]. There was also no difference in total FB on ECMO days 1–5 in patients with no change in functional outcome as reflected by the Pediatric Outcome Performance Category (POPC) score vs. those who had worsening in POPC score ≥2 at hospital discharge [median 98 ml/kg (IQR 18–267) vs. median 130 ml/kg (IQR 13–252), *p* = 0.91]. Subjects that required 50 ml/kg or more of blood products over the initial 5 days of ECMO support had an increased rate of mortality with an odds ratio of 5.8 (95% confidence interval of 2.7–12.3; *p* = 0.048). Our study showed no association of the noted FB with survival after ECMO cannulation. This FB trend was also not associated with POPC at hospital discharge, MV duration, or ECMO duration. The amount of blood product administered was found to be a significant predictor of mortality.

## Introduction

Extracorporeal membrane oxygenation (ECMO) is considered an effective rescue therapy for severe respiratory or cardiac failure (1–3). Overall survival for pediatric ECMO has remained at ~50–60% (4). Large-volume intravenous fluid infusions are often required after ECMO initiation due to hemorrhage and ongoing capillary leak, and these infusions are also needed to minimize venous access insufficiency in order to target an appropriate ECMO flow rate (1, 2, 5–9). A significant positive fluid balance (FB) remains a common characteristic in ECMO patients (as a consequence of fluid infusions and blood product administration) and is managed with diuretics and hemofiltration (1, 4, 10, 11).

The detrimental effects of positive FB on adult and pediatric patient outcomes in acute lung injury (ALI) are widely recognized (1, 12–15, 15–19). In 2006, the ARDS network published results of a randomized clinical trial comparing conservative vs. liberal fluid strategy in adult patients with ALI (20). The results of this trial demonstrated that a conservative fluid management strategy resulted in improved lung function, fewer mechanical ventilation (MV) days, and shorter ICU length of stay (20). *Post-hoc* analysis in pediatric ALI patients demonstrated a similar association between positive FB and increased MV days as well as overall mortality (21). The Pediatric Acute Lung Injury and Sepsis Investigators (PALISI) network supported these findings in 2012 with a retrospective study of children with ALI that demonstrated an association of increasing positive FB by day 3 with decreased ventilator-free days (VFD) (22).

The effects of a positive FB on ECMO patients have been evaluated in a retrospective observational adult study which investigated the relationship between early positive FB and 90-day outcome. The authors found that a positive FB on ECMO day 3 was an independent predictor of 90-day mortality (1). As mentioned above, a large volume of blood product infusion due to hemorrhage could result in fluid overload. Hemorrhagic events, assessed by the total of red blood cell units received during ECMO, were also shown to be associated with hospital mortality in adult patients (23). A pediatric single-center study of 128 patients who received extracorporeal life support (ECLS) demonstrated a decrease in mean body weight from 9 ± 2% over dry weight to 4 ± 2% in survivors. Non-survivors, however, demonstrated an increase in mean body weight from 25 ± 5% over dry weight to 35 ± 7% (24). A study in pediatric patients who received continuous renal replacement therapy (RRT) while on ECMO showed a lower fluid overload in survivors compared with non-survivors (24.5 vs. 38%, *p* = 0.006) (25). Gorga et al. noted that severe fluid overload was very common at CRRT initiation when on ECMO and Selewski et al. noted that fluid overload is a potential target to improve outcomes in pediatric patients on ECMO (26, 27). The specific effect of positive FB, its association with hemorrhage, and its correlation with the days on ECMO in pediatric patients remains unknown. Our objective was to evaluate if daily positive FB in pediatric intensive care unit (PICU) patients on ECMO was associated with increased morbidity or mortality. We also sought to evaluate if bleeding score and blood product administration differed in survivors vs. non-survivors.

## Materials and Methods

This was a multicenter retrospective cohort study of data from the deidentified PEDiatric ECMO Outcomes Registry (PEDECOR). The PEDECOR database is a national registry with data on patients who received mechanical support from ECMO at nine pediatric centers. The project was determined as not human research by the Human Research Protection Program/Institutional Review Board at Children's Wisconsin.

All patients entered into the PEDECOR database from 2014 to 2017 were included. These patients received either venoarterial ECMO (VA-ECMO) or venovenous ECMO (VV-ECMO). Patients with undocumented mortality or missing fluid balance on days 1–3 were excluded from the analysis.

Data extracted from the PEDECOR database included the following: pre-ECMO variables such as patient demographics, admission weight, preadmission details, indication for ECMO, Pediatric Index of Mortality (PIM) 2 score, and Pediatric Risk of Mortality (PRISM) III score and ECMO variables such as fluid balance on ECMO days 1–5, oxygenation index (OI) at cannulation, RRT, bleeding score, blood product transfusion volume on days 2 and 3 of ECMO, MV days, and ECMO days. The bleeding score was defined in the PEDECOR database on the following scale: 0 = no bleeding, 1 = mild bleeding, 2 = moderate bleeding, 3 = severe bleeding, and 4 = catastrophic bleeding. Further description of the bleeding score is available in Supplement 1. The blood transfusion requirements on day 1 were not included due to differences in reporting of blood products (at the different centers). Some centers included the blood used to prime the ECMO circuit at initiation of support, whereas others did not. The primary outcome was mortality at hospital discharge. Secondary outcomes included MV days, ECMO days, and morbidity as reflected by the change in functional outcome by an increase in the Pediatric Overall Performance Category score (POPC) by ≥2 from baseline to PICU discharge. The POPC score change was chosen as it has been shown to represent a change in global morbidity with moderate and severe deficits for the score predicting increased length of stay between 30 and 40% (28, 29).

The association between daily FB on ECMO days 1–5 with mortality or change in POPC score was analyzed by Mann–Whitney test. Patients were further grouped into those who were placed on VA-ECMO or VV-ECMO. Patient variables between ECMO survivors and non-survivors were compared including PIM/PRISM scores, OI at the time of cannulation, MV days, ECMO days, and aggregate volume of blood products transfused. Pearson's test was used to analyze the correlation between total FB against MV duration or ECMO days. Analysis was performed using SPSS v. 24 software. A two-tailed *p*-value of < 0.05 was considered to be statistically significant. A multivariable analysis of mortality was performed using logistic regression that controlled for subject age, PRISM III score, ECMO type, and blood product administration per kilogram of body weight.

## Results

**Figure 1:**
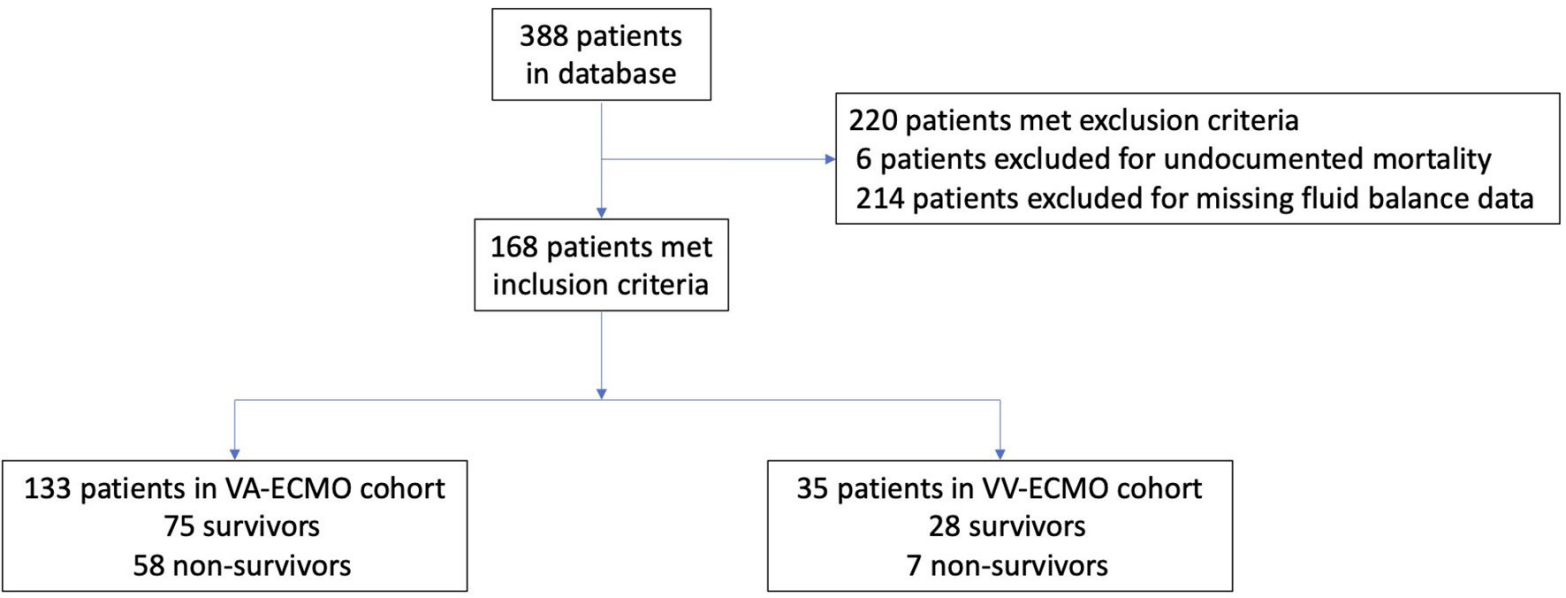
Screened patients and outcomes.

A total of 388 patients were entered into the PEDECOR database at the time of data request, of which 168 subjects met the study criteria (Figure 1). Six patients were excluded for undocumented mortality and 214 were excluded for missing fluid balance data in the first 3 days on ECMO. There were 103 survivors and 65 non-survivors. There were a total of 133 patients (75 survivors and 58 non-survivors) that received VA-ECMO and 35 patients (28 survivors and 7 non-survivors) that received VV-ECMO. Table 1 includes the characteristics of our patients. A total of 107 patients received ECMO for a primary cardiac indication and 55 patients received ECMO for a primary respiratory indication (six patients did not have a primary diagnosis entered). Only eight patients (4.5%) had a malignancy-related diagnosis (four in the VA-ECMO and four in the VV-ECMO cohort). Of these patients with a malignancy-related diagnosis, there was one non-survivor in the VA-ECMO cohort and one non-survivor in the VV-ECMO cohort.

**Table 1:** Comparison of risk factors and outcomes between survivors vs. non-survivors.

	**All patients** ** (*N* = 168)**	**Survivors** ** (*N* = 103)**	**Non-survivors** ** (*N* = 65)**	***p*-value**
Age (years)^a^	0.67 (0.05–6.4)	0.67 (0.04–3.5)	0.67 (0.06–9)	0.465
Weight (kg)^a^	7.3 (3.4–21.7)	7.4 (3.5–14.3)	6.8 (3.3–27.7)	0.857
Primary cardiac indication for ECMO^b,c^	107 (63.7%)	63 (58.9%)	44 (41.1%)	0.161
Primary respiratory indication for ECMO^b,c^	55 (32.7%)	38 (69.1%)	17 (30.9%)	0.163
Fluid balance on day 1 (ml/kg)^a^	56.5 (13.3–92.7)	57.1 (18.5–81.7)	53.9 (9.6–118.7)	0.645
Fluid balance on day 2 (ml/kg)^a^	32.3 (3.2–74.1)	28.7 (2.9–67.9)	10.1 (4.7–88.4)	0.374
Fluid balance on day 3 (ml/kg)^a^	7.2 (−22.1 to 51.6)	7.1 (−17.9 to 55.1)	10.1 (−26.9 to 47.3)	0.507
Fluid balance on day 4 (ml/kg)^a^	9.8 (−25.4 to 32.4)	8.8 (−30.1 to 31.8)	20.3 (−21 to 52.5)	0.313
Fluid balance on day 5 (ml/kg)^a^	1.4 (−20.5 to 28.6)	1.7 (−22.7 to 21)	1.2 (−20.3 to 38)	0.585
Total fluid balance (ml/kg)^a^	97.4 (16.9–241.6)	90 (18–208.5)	139.7 (11.2–300.6)	0.334
First 3-day total fluid balance (ml/kg)^a^	94.5 (13.2–196.7)	93.6 (18.1–185.3)	98.3 (8–241.2)	0.908
First 4-day total fluid balance (ml/kg)^a,d^	145.8 (13.8–227.2)	93.5 (36.3–195) (*N* = 90)	119.4 (−15.9 to 317.8) (*N* = 53)	0.480
Five-day total fluid balance (ml/kg)^a,d^	143.4 (11.4–234.6)	76.8 (21.7–122.7) (*N* = 74)	110.7 (−72.4 to 407.8) (*N* = 37)	0.804
PRISM^a^	14 (8–21.5)	14 (9–21)	14 (6.5–22.5)	0.763
OI^a^	24 (8.5–49)	32 (11.3–56.3)	16 (7–34)	0.091
MV^a^ days	18 (8–39.8)	18 (9.5–37.5)	17 (6–45)	0.377
ECMO days^a^	5 (3–8)	5 (3–8)	4 (2.5–9)	0.606
RRT^b^	85 (50.6%)	51 (49.5%)	34 (52.3%)	0.921
Change in POPC from baseline^a^	1 (0–4)	0 (0–1)	4 (3–5)	<0.001
Bleeding score^a^	1 (1–2)	1 (1–2)	2 (1–3)	0.012
Aggregate volume of blood products transfused on days 2 and 3 (ml/kg)^a^	58.6 (30.2–101.6)	50.1 (22.6–84.8)	81.7 (43.6–139.4)	0.001

*PIM, Pediatric Index of Mortality; PRISM, Pediatric Risk of Mortality; OI, oxygenation index; MV, mechanical ventilation; ECMO, extracorporeal membrane oxygenation; RRT, renal replacement therapy; POPC, Pediatric Outcome Performance Category*.

a *Reported as median and interquartile ranges (IQR) in parenthesis*.

b *Reported as number and percentage in parenthesis*.

c *Six patients did not have primary diagnosis data available*.

d *Reported data in patients when available on days 4 and 5*.

**Figure 2:**
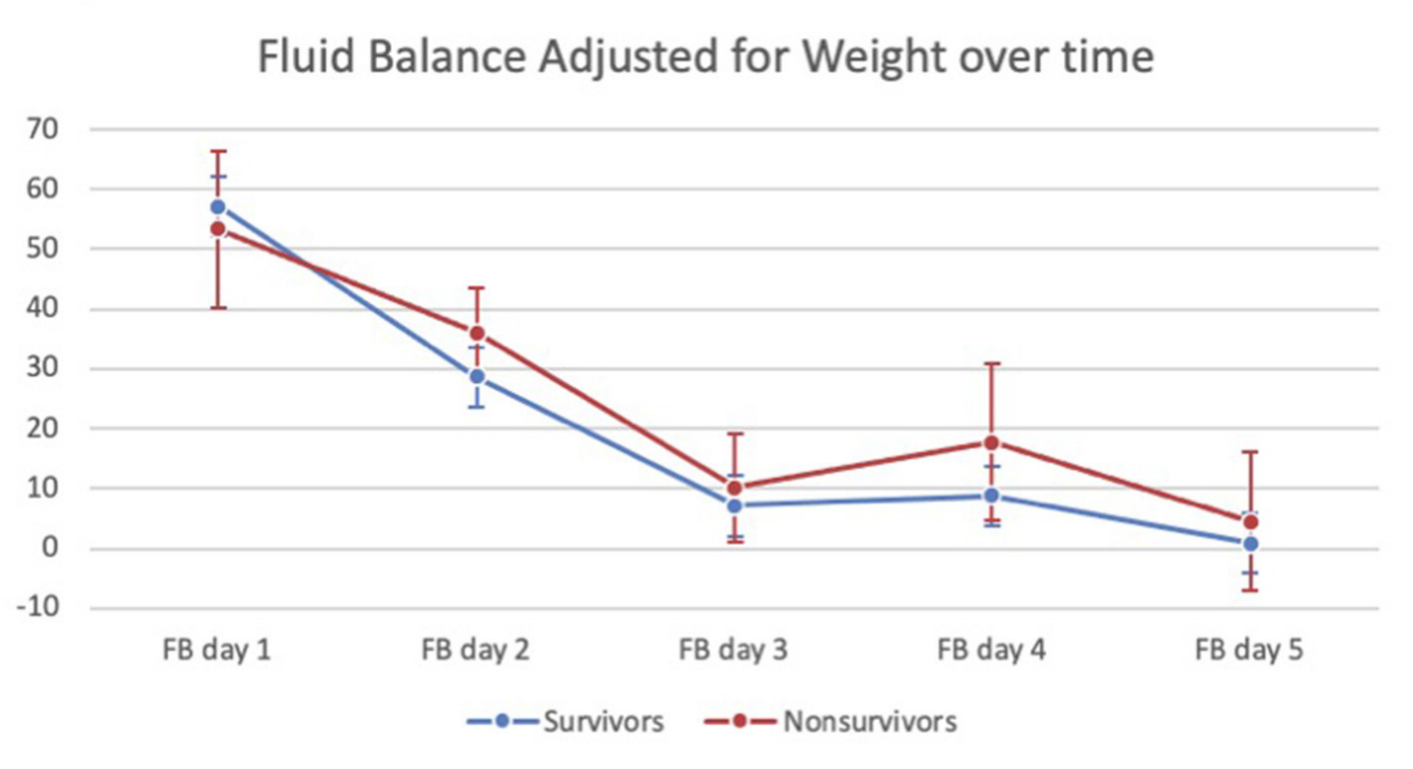
Fluid balance (adjusted for patient weight) in survivors vs. non-survivors over the first 5 days of ECMO.

Univariate analysis showed no significant difference for the entire cohort in total FB on ECMO days 1–5 between survivors and non-survivors [median 90 ml/kg (18–208.5) for survivors vs. median 139.7 ml/kg (IQR 11.2–300.6) for non-survivors, *p* = 0.334] (Table 1). Analysis of the VA-ECMO cohort showed no significant difference in total FB on ECMO days 1–5 [median 92.5 ml/kg (IQR 16.5–211) for survivors vs. median 134.6 ml/kg (IQR 12.3–316.9) for non-survivors, *p* = 0.364] (Table 2). Analysis of the VV-ECMO cohort also demonstrated no significant difference in FB on ECMO days 1–5 [median 74.7 ml/kg (IQR 23.4–177) for survivors vs. median 156.9 ml/kg (IQR −68.8 to 279.5) for non-survivors, *p* = 0.762] (Table 3). No association with mortality was found when the FB was compared on each day, as well as the total FB on ECMO days 1–3. The FB over the first 5 days, in survivors vs. non-survivors, is shown in Figure 2. The FB over the first 5 days by age categories is shown in Table 4.

**Table 2:** Comparison of risk factors and outcomes between survivors vs. non-survivors in the VA-ECMO (venoarterial extracorporeal membrane oxygenation) cohort.

	**Survivors (*N* = 75)**	**Non-survivors (*N* = 58)**	***p*-value**
Age (years)^a^	0.23 (0.03–1.45)	0.69 (0.05–6.9)	0.108
Weight (kg)^a^	5 (3.3–10.7)	7.2 (3.3–25.9)	0.351
Fluid balance on day 1 (ml/kg)^a^	59.3 (20.9–83.1)	53.9 (7.1–118)	0.781
Fluid balance on day 2 (ml/kg)^a^	31.8 (2.9–70)	36.9 (6.7–87.7)	0.723
Fluid balance on day 3 (ml/kg)^a^	4.6 (−22.2 to 57.4)	8.2 (−42.9 to 45.9)	0.534
Fluid balance on day 4 (ml/kg)^a^	3.2 (−39 to 31.4)	17.7 (−22.2 to 44.8)	0.213
Fluid balance on day 5 (ml/kg)^a^	0.78 (−29 to 19.8)	0.93 (−20.5 to 39.3)	0.611
Total fluid balance (ml/kg)^a^	92.5 (16.5–211)	134.6 (12.3–316.9)	0.364
First 3-day total fluid balance (ml/kg)^a^	99.8 (35.3–189)	101.3 (8.2–239)	0.939
First 4-day total fluid balance (ml/kg)^a,b^	98.9 (37.5–208.3) (*N* = 62)	117.5 (−6.9 to 320.1) (*N* = 48)	0.754
Five-day total fluid balance (ml/kg)^a,b^	94.4 (16.9–184.5) (*N* = 48)	104.4 (−74.2 to 424.2) (*N* = 32)	0.768
PRISM^a^	15 (9–21)	14 (7–22)	0.561
OI^a^	13.5 (6.3–43.5)	14 (7–32)	0.869
MV^a^ days	15.5 (8–32.8)	18 (6–47.5)	0.959
ECMO days^a^	4 (2–6)	4 (2.8–8)	0.798
RRT^c^	32 (42.7%)	27 (46.6%)	0.741
Change in POPC from baseline^a^	0 (0–1)	4 (3–5)	<0.001
Bleeding score^a^	1 (1–2)	2 (1–3)	0.041
Aggregate volume of blood products transfused on days 2 and 3 (ml/kg)^a^	58.7 (30.8–89.8)	84.6 (42.6–145.2)	0.029

*PIM, Pediatric Index of Mortality; PRISM, Pediatric Risk of Mortality; OI, oxygenation index; MV, mechanical ventilation; ECMO, extracorporeal membrane oxygenation; RRT, renal replacement therapy; POPC, Pediatric Outcome Performance Category; VA-ECMO, venoarterial extracorporeal membrane oxygenation*.

a *Reported as median and interquartile ranges (IQR) in parenthesis*.

b *Reported data in patients when available on days 4 and 5*.

c *Reported as number and percentage in parenthesis*.

**Table 3:** Comparison of risk factors and outcomes between survivors vs. non-survivors in the VV-ECMO (venovenous extracorporeal membrane oxygenation) cohort.

	**Survivors (*N* = 28)**	**Non-survivors (*N* = 7)**	***p*-value**
Age (years)^a^	2.4 (1.2–10.5)	0.1 (0.1–6.4)	0.558
Weight (kg)^a^	13 (8.4–30.7)	6.8 (4.2–58)	0.702
Fluid balance on day 1 (ml/kg)^a^	47.2 (10.4–75.4)	56.1 (12.4–135.3)	0.612
Fluid balance on day 2 (ml/kg)^a^	13.3 (0.1–42.2)	29.2 (−8.5 to 110.2)	0.643
Fluid balance on day 3 (ml/kg)^a^	11.1 (−6.9 to 43.7)	10.1 (−23.5 to 64.8)	0.856
Fluid balance on day 4 (ml/kg)^a^	11.7 (−9.3 to 31.9)	23.3 (−34.1 to 76)	1.000
Fluid balance on day 5 (ml/kg)^a^	2.6 (−18.2 to 28.6)	21.4 (−44.2 to 44.9)	0.856
Total fluid balance (ml/kg)^a^	74.7 (23.4–177)	156.9 (−68.8 to 279.5)	0.762
First 3-day total fluid balance (ml/kg)^a^	66.2 (4.9–163.3)	43.2 (−21.9 to 246.4)	0.920
First 4-day total fluid balance (ml/kg)^a,b^	69.3 (29–184) (*N* = 28)	135.5 (−68.4 to 419.6) (*N* = 5)	0.865
Five-day total fluid balance (ml/kg)^a,b^	74.7 (24.4–186.9) (*N* = 26)	156.9 (−112.6 to 464.6) (*N* = 5)	0.897
PRISM^a^	13 (6–24)	15.5 (4.5–26.8)	0.903
OI^a^	49 (23.8–68.5)	24.5 (17.5–42.3)	0.137
MV^a^ days	20 (12–50)	9 (5–64.8)	0.109
ECMO days^a^	7 (4.3–15.3)	2 (1–3)	0.952
RRT^c^	19 (67.9%)	6 (85.7%)	0.644
Change in POPC from baseline^a^	0 (0–1)	4.5 (4–5)	<0.001
Bleeding score^a^	1 (1–2)	2 (1–3)	0.236
Aggregate volume of blood products transfused on days 2 and 3 (ml/kg)^a^	31.7 (15.1–51.5)	73.9 (56.9–165)	0.018

*PIM, Pediatric Index of Mortality; PRISM, Pediatric Risk of Mortality; OI, oxygenation index; MV, mechanical ventilation; ECMO, extracorporeal membrane oxygenation; RRT, renal replacement therapy; POPC, Pediatric Outcome Performance Category; VV-ECMO, venovenous extracorporeal membrane oxygenation*.

a *Reported as median and interquartile ranges (IQR) in parenthesis*.

b *Reported data in patients when available on days 4 and 5*.

c *Reported as number and percentage in parenthesis*.

**Table 4:** Fluid balance by age.

	**Age (years)**				
	** <1 (*N* = 91)**	**1–5 (*N* = 34)**	**5–10 (*N* = 8)**	**>10 (*N* = 35)**	***p*-value**
Fluid balance on day 1 (ml/kg)^a^	74.3 (96)	38.3 (71)	84.1 (71)	53.6 (53)	0.122
Fluid balance on day 2 (ml/kg)	43.2 (72)	28.4 (46)	14.4 (40)	38.1 (42)	0.425
Fluid balance on day 3 (ml/kg)	27.9 (74)	9.7 (58)	−1.7 (24)	11.5 (44)	0.277
Fluid balance on day 4 (ml/kg)	21.8 (93)	14.5 (67)	0.5 (34)	5.2 (26.9)	0.714
Fluid balance on day 5 (ml/kg)	15.7 (82)	8.4 (59)	−12.3 (21)	−3.3 (26.8)	0.583
Total fluid balance (ml/kg)	173 (250)	92.7 (201)	89.5 (125)	105.4 (110)	0.142

a *Reported as mean and standard deviation in parenthesis*.

There were no differences in baseline POPC functional score, PRISM III and PIM2 illness severity scores, MV days, ECMO days, OI at the time of cannulation, or need for RRT between survivors and non-survivors in the entire cohort (Table 1). This was also true when the VA-ECMO and VV-ECMO cohorts were analyzed separately (Tables 2, 3). We further analyzed the fluid balance in patients who received RRT and those who did not. There was no significant difference in FB on ECMO days 1–5 [median 76.2 ml/kg (IQR −4.8 to 254.7) for those who received RRT vs. median 96.2 ml/kg (IQR −14.4 to 211) for those who did not receive RRT, *p* = 0.809]. There was no significant difference in FB on ECMO day 4 [median 12.6 ml/kg (IQR −28.1 to 35.5) for those who received RRT vs. median 9.8 ml/kg (IQR −23.5 to 30.7) for those who did not receive RRT, *p* = 0.741]. This was also true on day 5 [median 3.5 ml/kg (IQR −23.1 to 34) for those who received RRT vs. median 0.8 ml/kg (IQR −15.5–27.4) for those who did not receive RRT, *p* = 0.264].

The bleeding score was lower in survivors vs. non-survivors [median 1 (IQR 1–2) vs. median 2 (IQR 1–3), *p* = 0.012]. In the VA-ECMO cohort, the bleeding score was significantly lower in survivors [median 1 (IQR 1–2) vs. median 2 (IQR 1–3), *p* = 0.041] than in non-survivors. In the VV-ECMO cohort, the bleeding score was not significantly different in survivors [median 1 (IQR 1–2) vs. median 2 (IQR 1–3), *p* = 0.236] from non-survivors. Aggregate volume of transfused blood products on days 2 and 3 was significantly lower in survivors [median 50.1 ml/kg (IQR 22.6–84.8) vs. median 81.7 ml/kg (IQR 43.6–139.4), *p* = 0.01] compared with non-survivors in the entire cohort. This was also found to be true when data were compared between survivors vs. non-survivors in the VA-ECMO cohort [median 58.7 ml/kg (IQR 30.8–89.8) for survivors vs. median 84.6 ml/kg (IQR 42.6–145.2) for non-survivors, *p* = 0.029] and VV-ECMO cohort [median 31.7 ml/kg (IQR 15.1–51.5) for survivors vs. median 73.9 ml/kg (IQR 56.9–165) for non-survivors, *p* = 0.018].

There was no significant difference in total FB on ECMO days 1–5 in the 62 patients who had no change in POPC score [median 98 ml/kg (IQR 18–267) vs. median 130 ml/kg (IQR 13–252), *p* = 0.91] and for the 59 patients who had a change in POPC score ≥2 (at hospital discharge). This lack of association of FB and functional outcome persisted when the fluid balance was compared on each day, as well as the total FB on ECMO days 1–3. There was no significant correlation between the total FB on ECMO days 1–5 and MV days (*r*^2^ = 0.044, *p* = 0.1). There was also no significant correlation between the total FB on ECMO days 1–5 and ECMO days (*r*^2^ = 0.002, *p* = 0.546).

In the multivariable analysis, the amount of blood product administered was found to be a significant predictor of mortality. Subjects that required 50 ml/kg or more of blood products over the initial 5 days of ECMO support had an increased rate of mortality with an odds ratio of 5.8 (95% confidence interval of 2.7–12.3; *p* = 0.048).

## Discussion

Our study demonstrated no significant association of a slightly positive FB with a trend toward euvolemia by day 3 with survival to hospital discharge of pediatric ECMO patients. This was also true when we analyzed patients in the VA-ECMO and VV-ECMO cohorts separately. There was also no association of this trend with our secondary outcomes of MV days, ECMO days, or change in POPC score. Despite the non-survivors having a higher bleeding score and receiving more blood product transfusions than the survivors, the FB was still comparable. These data indicate that an early positive FB after ECMO cannulation is not associated with survival in this pediatric cohort.

The entire cohort showed similarity in severity of illness as measured by PIM score, PRISM score, OI score, and RRT utilization between survivors and non-survivors. There was no difference in positive FB between the survivors vs. non-survivors, while there was an association of mortality with blood product administration of >50 ml/kg. Adverse effects of blood product administration such as transfusion-associated lung injury and circulatory overload and their effect on clinical outcomes should be considered in this critically ill population.

We noticed a lack of association of this positive FB trend with outcomes. This is contrary to adult data by Schmidt et al. which demonstrated that a positive FB was an independent predictor of 90-day mortality (1). It should be mentioned that our study evaluated mortality dependent on survival to hospital discharge in comparison to the adult study. These data were also not concordant with the Swaniker study of pediatric ECMO patients cannulated for respiratory failure, which demonstrated a decrease in mean body weight over dry weight in survivors and an increase in mean body weight over dry weight in non-survivors (24). Our study assessed early FB (days 1–5) on ECMO and did not assess mean body weight over dry weight as in the Swaniker study. While mean body weight over dry weight is very helpful in assessing fluid overload from baseline, this information might not be readily available and may be inaccurate in patients with significant duration of illness prior to cannulation.

The trend of FB in our study and the adult study warrants discussion. Schmidt et al. demonstrated a lower FB on days 1–5 in survivors vs. non-survivors. Survivors in that study were noted to have an euvolemic median FB by day 3, while non-survivors appeared to have a median FB of >1,000 ml and achieved a euvolemic median FB by day 7. In our cohort, FB was not significantly different on days 1–5 in survivors vs. non-survivors. By day 3 on ECMO, our survivors and non-survivors had a median positive FB of <10 ml/kg. Survivors were noted to have an almost euvolemic median FB by day 5 albeit this was not statistically different from the median positive FB of 4.5 ml/kg in non-survivors. With the median ECMO days in the survivors and non-survivors being 4–5 days, we were unable to evaluate the trend beyond that time frame. It is unclear if small positive FB (<10 ml/kg) in both the survivors and non-survivors by day 3 in our study contributed to the lack of association of FB with mortality. It is unclear if the small positive FB was due to clinical vigilance and aggressive fluid management, and likely reflects the current fluid management practices among participating centers and resulted in difference in impact on survival. It is worth mentioning that RRT utilization was not associated with a lower positive FB. This was also true on days 4 and 5, where patients were more likely to have a negative FB.

The non-survivors in our cohort had a higher bleeding score and received more blood product transfusions and ultimately had a similar FB to our survivors. It is unclear if different protocols for bleeding interventions and differences in hemoglobin and platelet thresholds for blood product administration resulted in different amounts of blood product administration, resulting in a more tightly controlled FB in our pediatric study in comparison to the adult Schmidt study (since blood product administration was not discussed in the adult study).

Our study limitations are as follows. First, our results are reported from a database to which limited centers contribute which may limit generalizability. Like any retrospective study, our results are subject to confounding bias with conclusions implying association and not causation. The inclusion of multiple centers also introduces variability at the institutional level (such as timing of cannulation, protocol for fluid and blood product resuscitation on ECMO, and RRT initiation). Our study did not evaluate the impact of fluid balance prior to cannulation, which could also have a significant effect on outcomes and warrants evaluation. In addition, current practices of managing FB (such as fluid restriction, aggressive diuresis, and early dialysis) were not evaluated. Fluid management practice in the centers included in this study did not result in large positive FB by ECMO day 3. We were limited in our ability to compare pre-ECMO data and patient diagnoses (other than primary cardiac vs. respiratory indication for ECMO and identification of a malignancy-related diagnosis), and we had to exclude patients due to missing data which could have resulted in selection bias. Finally, the timing of initiation of RRT was not part of our study; however, it has to be mentioned that utilization of RRT was not associated with a lower FB.

## Conclusion

Our study showed no association of the noted FB with survival after ECMO cannulation in our pediatric population. Positive FB was comparable between survivors and non-survivors, despite non-survivors having more bleeding and requiring more blood product transfusions. This FB trend was also not associated with the decline in POPC at hospital discharge, longer ECMO days, or longer MV duration. The amount of blood product administered was found to be a significant predictor of mortality.

## Data Availability Statement

The raw data supporting the conclusions of this article will be made available by the authors, without undue reservation.

## Author Contributions

PR conceptualized the study, drafted the manuscript, and approved the final version of the manuscript. LC designed the study, drafted the initial manuscript, and approved the final version of the manuscript. NT, TP, and SH helped to conceptualize the study, reviewed/revised the manuscript, and approved the final version of the manuscript. All authors contributed to the article and approved the submitted version.

## Conflict of Interest

The authors declare that the research was conducted in the absence of any commercial or financial relationships that could be construed as a potential conflict of interest.

## Publisher's Note

All claims expressed in this article are solely those of the authors and do not necessarily represent those of their affiliated organizations, or those of the publisher, the editors and the reviewers. Any product that may be evaluated in this article, or claim that may be made by its manufacturer, is not guaranteed or endorsed by the publisher.
