# Supplementary material for: Association of Fluid Balance and Survival of Pediatric Patients Treated With Extracorporeal Membrane Oxygenation
Source: Front Pediatr. 2021 Sep 16;9:722477. doi: 10.3389/fped.2021.722477 (PMC8481698; doi:10.3389/fped.2021.722477)
Supplement: Supplementary file 1 [file Table_1.DOCX]

**Supplement 1:** Bleeding score scale as defined in the PEDiatric ECMO Outcomes Registry (PEDECOR) database

0 = Zero, no or minimal bleeding

a. Oozing from catheter sites or mucosa (oral/nasal) without any intervention besides gauze or minimal pressure

1 = Mild bleeding

a. Oozing from catheter sites (ECMO cannulae, central venous lines, arterial catheter, PIV, chest tube…) that require minor interventions (surgery consult, extra suture, topical antifibrinolytics, surgicel)

b. Oozing from oral and/or nasal mucosa that requires surgical consultation and minor interventions (packing, suturing, topical antifibrinolytics…)

2 = Moderate bleeding

a. Peritoneal bleed, hemothorax, pulmonary bleed, and/or hematuria that does not require surgical intervention

b. Documented blood loss > 1 ml/kg/hr (averaged over 4 hrs), but less than 4 ml/kg/hr (see score 3)

c. Clinical bleeding requiring a decrease in the anti Xa/ACT goal, increase platelet transfusion threshold, or immediate transfusion of platelet, fresh frozen plasma or packed red blood cells (not otherwise indicated by lab parameters)

d. Use of intravenous antifibrinolytic drugs for bleeding (aminocaproic acid or tranexamic acid)

3 = Severe bleeding

a. Peritoneal bleed, hemothorax, pulmonary bleed, and/or hematuria that requires surgical intervention

b. Documented blood loss > 4 ml/kg/hr (averaged over 4 hrs)

c. Heparin drip held (unless in preparation for planned surgical procedure or excessive heparin level/ACT)

d. Bleeding leading to hemodynamic changes that requires blood transfusion(s)

e. New CNS bleed not requiring discontinuation of ECMO

4 = Catastrophic bleeding

a. Bleed resulting in death or requiring discontinuation of ECMO
